# Structure-to-Efficacy Relationship of HPMA-Based Nanomedicines: The Tumor Spheroid Penetration Study

**DOI:** 10.3390/pharmaceutics12121242

**Published:** 2020-12-20

**Authors:** Júlia Kudláčová, Lenka Kotrchová, Libor Kostka, Eva Randárová, Marcela Filipová, Olga Janoušková, Jun Fang, Tomáš Etrych

**Affiliations:** 1 Institute of Macromolecular Chemistry, Czech Academy of Sciences, Heyrovskeho nam. 2, 162 06 Prague 6, Czech Republic; kudlacova@imc.cas.cz (J.K.); kotrchova@imc.cas.cz (L.K.); kostka@imc.cas.cz (L.K.); koziolova@imc.cas.cz (E.R.); filipova@imc.cas.cz (M.F.); janouskova@imc.cas.cz (O.J.); 2 Faculty of Pharmaceutical Science, Sojo University, Ikeda 4-22-1, Nishi-ku, Kumamoto 860-0082, Japan; fangjun@ph.sojo-u.ac.jp

**Keywords:** HPMA polymers, pirarubicin, tumor spheroids, penetration, cytotoxicity

## Abstract

Nanomedicines are a novel class of therapeutics that benefit from the nano dimensions of the drug carrier. These nanosystems are highly advantageous mainly within cancer treatment due to their enhanced tumor accumulation. Monolayer tumor cells frequently used in routine preclinical assessment of nanotherapeutics do not have a spatial structural architecture that allows the investigation of the penetration of nanomedicines to predict their behavior in real tumor tissue. Therefore, tumor spheroids from colon carcinoma C26 cells and glioblastoma U87-MG cells were used as 3D in vitro models to analyze the effect of the inner structure, hydrodynamic size, dispersity, and biodegradability of *N*-(2-hydroxypropyl)methacrylamide (HPMA) copolymer-based nanomedicines carrying anticancer drug pirarubicin (THP) on the penetration within spheroids. While almost identical penetration through spheroids of linear and star-like copolymers and also their conjugates with THP was observed, THP penetration after nanomedicines application was considerably deeper than for the free THP, thus proving the benefit of polymer carriers. The cytotoxicity of THP-polymer nanomedicines against tumor cell spheroids was almost identical as for the free THP, whereas the 2D cell cytotoxicity of these nanomedicines is usually lower. The nanomedicines thus proved the enhanced efficacy within the more realistic 3D tumor cell spheroid system.

## 1. Introduction

Pirarubicin (4-O’-tetrahydropyranyl doxorubicin, THP) is a potent anthracycline anticancer drug that suppresses topoisomerase II, inhibits DNA synthesis through intercalation into DNA [1], and induces apoptosis through the generation of reactive oxygen species [2]. Compared to its widely used analog doxorubicin (DOX), THP exhibits equal or slightly stronger cytotoxicity, less cardiotoxicity, and enhanced long-term antitumor efficacy in the treatment of human solid tumors [3]. To minimize its negative side effects and maximize its antitumor activity, various nano-sized drug delivery systems (DDS), also called nanomedicines, have been developed for the targeted delivery of THP to tumor tissue [4,5,6]. Among them, biocompatible hydrophilic HPMA copolymers have proved to be advantageous vehicles for THP as well as other cytotoxic agents since their conjugates with drugs exhibit substantially decreased systemic toxicity and are preferentially accumulated in solid tumors compared to other healthy organs due to the Enhanced Permeability and Retention (EPR) effect [7,8,9,10]. The general versatility of HPMA copolymers enabled the synthesis of various high molecular weight (HMW) polymer systems, e.g., diblock, grafted, branched, or star-like polymers in a huge range of hydrodynamic sizes from 10 to almost 100 nm [11]. Compared to their linear analogs, these HMW polymer nanomedicines accumulate in solid tumors with fenestrated blood vessels to a higher extent and exhibit increased efficiency of tumor therapy, i.e., the application of star-like polymer conjugates with THP led to 100% cured mice with murine sarcoma [10,12,13]. However, all the nanomedicines should be excretable after fulfilling their role in drug delivery. Recently, complex smart structures with the ability to disintegrate to smaller polymer subunits after drug transport were designed, such as multiblock or star-like HPMA copolymer systems prepared by polycondensation or grafting of linear HPMA polymers to dendritic core based on 2,2-bis(hydroxymethyl)propionic acid (bis-MPA). The hydrolytic cleavage of the polyester core in star polymers enabled easy elimination of the polymer carrier [14].

To guarantee the desired anti-cancer effect, the drug should be activated in the target tissue, which is mediated by the tumor stimuli-sensitive linker between the polymer backbone and anticancer drug enabling drug release. The appropriate drug release can be controlled by using different biodegradable spacers such as pH-sensitive hydrazone bond hydrolyzable in the acidic tumor microenvironment (pH = 6.0–7.0) and intracellularly in lysosomes (pH = 5.0–5.5), oligopeptide linkage (for example Gly-Phe-Leu-Gly sequence, GFLG) degradable by lysosomal proteases, hydrolytically unstable ester bond or reductively degradable disulfide [12,13,15,16].

Besides the tumor accumulation rate and controlled drug release, the tumor penetration, i.e., distribution throughout tumor tissue, and the uptake to tumor cells of polymer-bound drugs should be considered when designing the optimal DDS. The suitable tumor distribution predetermines the therapeutic effectiveness of polymer-drug conjugates, especially in the treatment of large-sized heterogeneous tumors. These aspects must be considered in the design of optimal drug-polymer conjugates to meet multiple requirements, such as stability in the blood, preferential accumulation in the target tissue, controlled release of drug from polymer, and finally the elimination of polymers from the body by glomerular filtration.

Although the most reliable way of verifying the therapeutic efficiency of novel polymer-drug conjugates is the employment of cost-intensive experimental animals [17], the amount of newly developed therapeutics and the principles of ethical animal use do not permit such extensive testing. Therefore, countless in vitro assays using two-dimensional (2D) cell cultures are utilized before in vivo tests. These well-established in vitro models with precisely specified parameters allow large scale experiments, however, they cannot clarify the ability of polymer therapeutics to overcome the biological barriers of tumor tissue and to penetrate the deeper parts of tumors which may differ diametrically for polymer systems of diverse structure and size. The similarities with in vivo solid tumors or metastasis can be approximated by three-dimensional (3D) tumor spheroids where every cell has a different metabolic profile depending on the distance from the surface of the spheroid. The cells inside are in a hypoxic region while surface cells are the most proliferative due to the sufficient supply of nutrients and oxygen similar to the cells near blood vessels in real tumor tissue [18]. These increasingly attractive models for mimicking the structure and microenvironment of tumors and metastasis can serve as a useful tool for fast and facile prediction of the therapeutic response of potential nanomedicines without the need to sacrifice animals [19]. Also, due to the micrometer size of tumor spheroids, the penetration of polymer therapeutics can be observed in time on the cellular level.

In our previous work, the biological activity of the polymer conjugates of diverse architecture with anticancer drugs, especially anthracyclines, was studied in detail in vitro using 2D cell lines or in vivo using mice [13,14,20,21]. Nevertheless, the in-depth understanding of the structure-to-efficacy relationship is missing and cannot be obtained easily using in vivo animal experiments due to the enormous number of samples, thus the 3D spheroid models could be used instead to clarify the potential of biodegradable star polymer conjugates compared to their linear analogs.

In the present study, we employed 3D spheroids of C26 colon carcinoma and U87-MG glioblastoma to study the penetration behavior of fluorescently labeled linear HPMA copolymers and star-like HMW polymer structures. Since the therapeutic effect of polymer conjugates should be influenced by the type of linkage between THP and the polymer, the THP-polymer conjugates with hydrazone bond were synthesized and their penetration into spheroids was also studied in detail. Also, the cytotoxic effect of various nanomedicines in comparison to free THP was evaluated in a conventional 2D monolayer cell model and 3D spheroid model to fully exploit the structural aspects of the nanomedicines onto the antitumor efficacy.

## 2. Materials and Methods

### 2.1. Materials

Methacryloyl chloride, 1-amino-2-propanol (AP), dichloromethane (DCM), 2,2′-azobis(isobutyronitrile) (AIBN), 4-cyano-4-thiobenzoylsulfanylpentanoic acid, ethylacetate, acetone, diethyl ether, 4,5-dihydrothiazole-2-thiol, dimethylformamide (DMF), *N*,*N*′-dicyclohexylcarbodiimide (DCC), dimethyl sulfoxide (DMSO), *N*,*N*-diisopropylethylamine (DIPEA), dimethylacetamide (DMA), trifluoroacetic acid (TFA), acetic acid, tert-butyl alcohol (t-BuOH), sodium chloride (NaCl), methanol, sodium acetate, sodium cyanoborohydride,2,4,6-trinitrobenzene-1-sulfonic acid (TNBSA) were purchased from Sigma-Aldrich Inc., 2,2′-azobis(4-methoxy-2,4-dimethylvaleronitrile) (V70) was purchased from FUJIFILM Wako Pure Chemical Corporation, Neuss, Germany. Pirarubicin (THP) was purchased from Meiji Seika, Japan. Dy-395XL-NHS ester was purchased from Dyomics GmbH, Germany. LH-20 was purchased from Cytiva. Bis-MPA-16-ammonium dendron with acetylene core (G4), bis-MPA-24-ammonium dendrimer with trimethylolpropane (G3) were purchased from Polymer Factory, Sweden. Poly(amidoamine) (PAMAM) dendrimer G3 with ethylenediamine core (32 primary amines on the surface) was purchased from Sigma-Aldrich. RPMI-1640 medium, 3-(4,5-dimethylthiazol-2-yl)-2,5-diphenyltetrazolium bromide (MTT) were purchased from Wako Pure Chemical, Osaka, Japan, fetal bovine serum (FBS) was purchased from Nichirei Biosciences Inc., Tokyo, Japan. All chemicals and solvents were analytical grade.

### 2.2. Methods

#### 2.2.1. Synthesis of Monomers and Functionalized Chain Transfer Agent

*N*-(2-Hydroxypropyl)methacrylamide (HPMA) was synthesized by the reaction of freshly distilled methacryloyl chloride with 1-amino-2-propanol in dichloromethane (DCM) [22]. Boc-protected *N*-(*tert*-butoxycarbonyl)-*N*’-(6-methacrylamidohexanoyl)hydrazine (Ma-Acap-NHNH-Boc) was prepared as previously described in ref. [16]. The chain transfer agent (CTA) 2-cyano-5-oxo-5-(2-thioxo-1,3-thiazolidin-3-yl)pentan-2-yl ethyl carbontrithioate (TTC-TT) was synthesized as described previously [23].

#### 2.2.2. Synthesis of Linear Copolymer Precursors

Linear copolymer precursors (**L1**, **L2, L3**) were synthesized by reversible addition-fragmentation chain transfer (RAFT) copolymerization of HPMA with Ma-Acap-NHNH-Boc using V-70 as an initiator and TTC-TT as a CTA. The molecular weights of the copolymers were controlled by using different molar ratios of monomers:CTA:initiators, 280:2:1 for **L1**, 560:2:1 for **L2**, and 580:2:1 for **L3**. The molar ratio of HPMA:Ma-Acap-NHNH-Boc in the reaction mixture was 92:8. Monomer HPMA was dissolved in t-BuOH and transferred into a glass ampoule. The second comonomer Ma-Acap-NHNH-Boc, V-70, and TTC-TT were dissolved in anhydrous DMA and added to the HPMA solution so that a maximum of 10% of DMA was in the final volume of mixture t-BuOH/DMA and the monomer concentration in solution was 0.8 mol·dm^−3^. As an example, **L1** was synthesized as follows, HPMA (4 g, 27.93 mmol) was dissolved in 34.2 mL of t-BuOH and Ma-Acap-NHNH-Boc (761 mg, 2.43 mmol), TTC-TT (82.5 mg, 0.22 mmol) and V-70 (33.5 mg, 0.11 mmol) dissolved in 3.8 mL DMA, both solutions were added to the ampoule and bubbled with argon for 10 min, then the ampoule was flame sealed. The polymerization was conducted at 30 °C for 72 h. The products were isolated by precipitation into a mixture of acetone and diethyl ether (1/1 *v*/*v*), filtered, and dried under vacuum. The product was dissolved in dried methanol and precipitated into a mixture of acetone/diethyl ether (1/1 *v*/*v*) again, filtered, washed with diethyl ether, and dried under vacuum.

In the next step, the trithiocarbonate end groups (TTC) were removed by the reaction with 20 wt% AIBN in DMA (10% *w*/*w* solution of polymer) at 80 °C for 3 h. The polymers were isolated and purified by precipitation into ethyl acetate, washed with diethyl ether, dried under vacuum, and subsequently re-precipitated from methanol into the ethyl acetate, washed with diethyl ether and dried under vacuum. The terminal reactive thiazolidine-2-thione (TT) groups on linear copolymer **L1** were blocked by adding 3-amino-2-propanol (AP) to the polymers dissolved in DMSO. The remaining AP was removed by a Sephadex LH-20 column with methanol as the mobile phase. The copolymers were dissolved in water after the evaporation of methanol and freeze-dried.

The linear polymer precursor **L4** was prepared by free-radical copolymerization of HPMA (100 mg, 0.70 mmol) with Ma-Acap-NHNH-Boc (19.03 g, 0.06 mmol) using AIBN (19 mg, 0.12 mmol) as an initiator in DMSO (0.74 mL) at 60 °C for 6 h under argon according to the previously described procedure [24]. The copolymer was isolated as in the case of the copolymers **L1** described above. All linear polymer precursors and their characteristics are summarized in Table 1.

#### 2.2.3. Synthesis of Star-Like Copolymer Precursors

The star-like polymer precursors (**S1**, **S3**) were prepared by aminolysis of TT groups of semitelechelic HPMA copolymers (**L3**) with amino groups of bis-MPA dendron or PAMAM dendrimer in DMA in presence of DIPEA for 4 h [14]. The star-like polymer **S2** was prepared by the same procedure using a bis-MPA dendrimer core and linear polymer precursor **L2**. The example for the synthesis of **S1** is as follows: bis-MPA dendron (3.72 mg, 0.78 mmol) was dissolved in DMA (0.5 mL) in the presence of DIPEA (4.85 mg, 37.5 mmol). After 40 min, the polymer **L3** (250 mg, 7.0 mmol TT groups) was dissolved in DMA (4.25 mL) and added to the bis-MPA dendron solution. The reaction mixture was stirred at LT (25 °C). After 4 h, the free amine groups on the cores were blocked by adding acetyl acid-TT. The product was isolated by precipitation into ethyl acetate, filtered, and dried. The star-like copolymers were purified from the unbound linear polymers by size exclusion chromatography using a Sephacryl S-300 column with 0.15 mol·dm^−3^ NaCl solution as the eluent (UV detection at 230 nm), then desalted and freeze-dried.

The polymer precursors **L1**, **L2**, **L4**, **S1,** and **S2** with free hydrazide groups were obtained by removing the protective Boc groups from the hydrazides in water at 100 °C for 1 h and isolated by freeze-drying [23]. The deprotection of the Boc-protected groups in the copolymer precursor **S3** was performed in concentrated TFA (10 wt% solution of polymer) for 15 min, followed by precipitation into diethyl ether and drying under vacuum. The final precursor **S3** was purified by double precipitation from methanol into diethyl ether, filtered, and dried under vacuum. Table 1 includes all star-like polymer precursors.

#### 2.2.4. Polymer Conjugates with THP

The polymer conjugates with THP attached via a hydrazone bond (Table 2) were prepared by the reaction of hydrazide groups respective copolymers with the keto group of THP in methanol in the dark with the addition of concentrated acetic acid as follows: **L1** polymer with free hydrazides (20 mg) and THP (2.3 mg) were dissolved in 0.2 mL methanol with 8 µL of acetic acid and left to react for 24 h in the dark. The polymer-drug conjugates were purified from unbound THP by double precipitation into ethyl acetate from methanol, filtered, and dried under vacuum.

#### 2.2.5. Polymer Conjugates with Dy-395XL

The fluorescently labeled copolymers were prepared by the conjugation of polymer precursors (Table 1) containing hydrazide groups with *N*-hydroxysuccinimide ester (NHS ester) of the fluorescent dye Dy-395XL (ex/em 396/572 nm) forming a hydrazide bond stable in a biological environment. The reaction was performed in methanol and DIPEA in the dark for 24 h. Specifically, 10 mg of polymer precursor **L1** was mixed with 0.3 mg of Dy-395XL-NHS ester in 100 µL of methanol and 2 µL of DIPEA. The fluorescent polymer conjugates were purified from an unbound fluorescent dye by gel permeation chromatography on a Sephadex LH-20 column in methanol and subsequent gel filtration on a Sephadex G25 column in water (PD10 column). The lyophilized conjugates (Table 3) were then used to investigate polymer penetration into the spheroids.

#### 2.2.6. Characterization of Polymer Precursors and Conjugates

The number-average molecular weight (*M_n_*), weight-average molecular weight (*M_w_*), and the dispersity (*Ð*) of the polymer precursors and conjugates (in the concentration of 3 mg·mL^−1^) were determined using size exclusion chromatography (SEC) on an HPLC Shimadzu system equipped with an SPD-M20A photodiode array detector (Shimadzu, Kyoto, Japan), an Optilab^®^rEX differential refractometer (Wyatt Technology, Galeta, CA, USA) and a multi-angle light scattering DAWN^®^ HELEOS II (Wyatt Technology, Galeta, CA, USA) detector using 0.3 mol·dm^−3^ sodium acetate buffer at pH 6.5 and methanol (20/80% *v/v*) as the mobile phase. TSKgel G3000SWxl or G4000SWxl column were used. The refractive index increment *dn/dc* of 0.167 mL/g was used for the determination of the weight-average molar mass *M_w_*, number-average molar mass *M_n_* and dispersity of polymers in ASTRA software (Wyatt Technology, Galeta, CA, USA).

The size of polymer precursors was analyzed via Dynamic Light Scattering (DLS) with Nano-ZS instrument Zetasizer (ZEN3600, Malvern, UK) by dissolving the polymer in 0.150 mol·dm^−3^ NaCl (3 mg/mL). DLS measurements were performed at a wavelength of 632.8 nm with a detection angle of 173° at 25 °C. The diameter sizes were the mean (by number) of ten measurements.

The terminal TT-group content in linear copolymer precursors was determined by UV-vis absorption spectroscopy in methanol by using ε = 10,800 dm^3^·mol^−1^·cm^−1^ at 305 nm and used for calculations of the polymer functionality. The hydrazide content after removal of the Boc-protected groups was determined spectrophotometrically at 500 nm using 2,4,6-trinitrobenzene sulfonic acid (TNBSA) according to the procedure described in ref. [23].

The amount of THP bound to polymer precursors was calculated from the absorption spectra of conjugates in methanol using ε = 11,200 dm^3^·mol^−1^·cm^−1^ at 490 nm. Similarly, the content of Dy-395XL in conjugates was determined from the value of the maximum absorbance of the absorption peak of fluorescently labeled polymer conjugate dissolved in water using ε = 20,000 dm^3^·mol^−1^·cm^−1^.

#### 2.2.7. Preparation of Spheroids and Uptake of Polymer Conjugates

The murine colon carcinoma cell line C26 was obtained from Riken Cell Bank (deposited from Tohoku University) and cultivated in RPMI-1640 medium (FUJIFILM Wako Pure Chemical Corporation, Osaka, Japan) containing 10% FBS and 100 units/mL penicillin-streptomycin under a humidified atmosphere of 5% CO_2_ at 37 °C. The cells (2 × 10^5^) were seeded in 14 cm^2^ ultra-low attachment cell dishes (Corning Inc., Corning, NY, USA). After 6 days of cultivation, formed cell spheroids were transferred into 9.6 cm^2^ glass-bottom culture dishes. The THP-polymer conjugates (Table 3) or HPMA polymers labeled with Dy-395XL in a final concentration of 5 µg drug or dye was applied for 6 h or 24 h, respectively, and the drug and fluorophore were visualized by confocal laser fluorescence microscopy (Nikon TE2000U, Nikon Solutions Co., Ltd., Tokyo, Japan) with the excitation wavelength at 488 nm and emission wavelength at 570−640 nm for THP, and Ex408 nm/Em570−640 nm for Dy-395XL.

The human glioblastoma cell line U87-MG was obtained from ATCC (ATCC, LGC Standards Sp. Z.o.o., Poland) and cultured in Eagle’s minimum essential medium (EMEM, Sigma-Aldrich, Prague, Czech Republic) containing 10% FBS and 100 units/mL penicillin-streptomycin under a humidified atmosphere of 5% CO_2_ at 37 °C. After reaching ~80% confluence, the cells were harvested with 0.05% trypsin/EDTA solution for 3 min and centrifuged at 580× *g* for 2 min. After removing the medium, the cells were passaged three times through a syringe with a 25 G needle in a small amount of fresh medium to obtain a single-cell suspension. To initiate spheroid growth, the cells were seeded in complete EMEM medium at a density of 9.6 × 10^3^ cells/cm^2^ into ultra-low attachment 6-well plates (Corning Inc., Corning, NY, USA) and cultured in the presence of 5% CO_2_ at 37 °C for three days. The performed spheroids were treated with 5 µg/mL of THP or dye equivalent of THP-polymer conjugates or fluorescently labeled polymers for 6 h or 24 h, respectively. The laser scanning confocal microscope (Olympus IX83, Olympus, Prague, Czech Republic) with FV10-ASW software was used to observe the fluorescence of THP and polymers. The drug THP was excited at 488 nm and emitted light was detected through the 500–600 nm filter. The dye was excited at 405 nm and emitted light was detected through a 525–625 nm filter.

#### 2.2.8. Cytotoxicity Assay

Mouse colon cancer C26 cells were maintained in RPMI-1640 supplemented with 10% FBS in 5% CO_2_/air atmosphere at 37 °C. For the cytotoxicity study, C26 cells were seeded in a 96-well plate (3000 cells/well) and incubated overnight before the addition of THP-polymer conjugates. The cells were incubated for 48 h and viable cells were determined by the MTT assay [25].

In a separate experiment, the cytotoxicity of THP-polymer conjugates against C26 spheroid was examined. In brief, C26 cells (20,000 cells/well) were seeded in 96-well ultra-low attachment well plates (Corning Inc., Corning, NY, USA) and cultured for 4 days until spheroids formed. Then, THP-polymer conjugates were added and the cells were incubated for 72 h, viable cells were determined by the MTT bromide assay [25].

## 3. Results and Discussion

The therapeutic efficacy of HPMA-based polymer nanomedicines is strongly dependent not only on its sole structure and size but is also affected by the tumor type, e.g., tumor growth rate, fenestration of the tumor vasculature, and overall penetrability of the polymer system in the tumor tissue. Recently, the beneficial effects of linear and star-like nanomedicines were demonstrated in solid tumor treatment [14,26]. The present study investigated the structure-to-efficacy relationship of anticancer properties of various HPMA-based polymer nanomedicines evaluated in 3D-tumor cell spheroids. In this work, spheroids prepared from colon carcinoma C26 tumor cells and glioblastoma U87-MG cells were employed in this study as in vitro 3D solid tumor models. Six HPMA polymer systems differing in structure, size, and dispersity were synthesized and labeled with fluorescent dye Dy-395XL to monitor their distribution in the cross-section of the spheroids. The effect of THP-polymer conjugate structure, size, and dispersity on the distribution of THP within the spheroids and the cytotoxic activity was also studied and compared with free THP.

### 3.1. Synthesis of HPMA Polymer Conjugates

Linear polymer systems differing in molecular weight, hydrodynamic size, and dispersity and HMW star-like polymer systems differing in the core and biodegradability were synthesized to compare the influence of the polymer structure, dispersity, and hydrodynamic size on the polymer distribution in tumor spheroids and their cytotoxicity against tumor cells and its spheroids. The linear copolymer precursors prepared by controlled RAFT polymerization (**L1**, **L2**) differing in the molecular weight had a narrow distribution of molecular weights (*Đ* < 1.12) when compared with dispersity of **L4** polymer (*Đ* = 2.09) synthesized by classical radical polymerization (Table 1, Figure S1).

Three types of star polymer precursors differing in the core type were prepared. Biodegradable bis-MPA cores and stable PAMAM dendrimer core were used to study the effect of biodegradability on the biological behavior of the polymers. A controlled grafting-to approach was employed using the polymer precursors **L2** and **L3** with high functionality of end-chain TT groups (Table 1), which were used for covalent attachment of precursors to amino groups on dendritic cores. The star polymer precursors **S1**, **S2,** and **S3** with a molecular weight in the range 300–340 kg·mol^−1^ and hydrodynamic size 21–23.7 nm were prepared, see Figure S1. The remaining free amine groups on the cores were blocked with an acetyl-TT to avoid the potential cytotoxic effect on the cells. The star precursors were freed of unbound linear precursors and the final star polymer had low dispersity (*Đ* < 1.05). The removal of the Boc-protection groups did not cause significant changes in *M_w_* and *Đ*. After deprotection, the hydrazides content of the HPMA copolymer chains was more than 6.8 mol% which was sufficient to bind both the anticancer agent THP or the fluorescent dye.

The content of THP attached via pH-sensitive hydrazone bond, in range 7–10 wt%, was selected based on our previous studies with comparable THP-polymer conjugates [10]. The conversion of the conjugation reaction reached nearly 100%, and the attachment of the drug had no significant influence on the molecular weight or the polydispersity of the polymer conjugates. We were not able to determine the exact *D*_h_ size values as the fluorescence of Dox influenced the scattering intensity during the DLS measurements. Nevertheless, based on recent results we can conclude that the hydrodynamic size in the solution after drug attachment is not increasing significantly [8].

All samples were labeled with Dy-395XL fluorescent dye via a stable hydrazide bond to follow the penetration of polymer-carriers in the spheroids. The attachment of the dye to copolymers did not cause any significant change in the size of the copolymers as determined by the RI detector. The direct determination of molecular weight was not possible due to the interference of fluorescence with the detection system of the MALS detector. The content of dye in polymers (Table 3) was sufficient for the determination of the fluorescence in all spheroid studies.

### 3.2. Spheroid Penetration Studies

Confocal microscopy was employed to evaluate the penetration of polymer-carriers into spheroids formed from C26 or U87-MG cells as a model of nanomedicine penetration from blood capillaries into the surrounding avascular tumor tissue. The advantages of these models are their good reproducibility and homogeneity. The experiments were performed using the spheroids grown to 250–300 µm size (C26 cell line) or 450–550 µm size (U87-MG cell line) suitable for the microscope observations to compare the nanomedicine distribution in spheroids of different type and size. We chose the colon cancer cell line C26 and U87-MG glioblastoma cell line because the treatment of these types of cancer is highly important from the general point of view. Despite current advances in treatment especially of early diagnosed colorectal cancers, this type of cancer still belongs to one of the most common causes of cancer deaths in the U.S. In the case of glioblastoma tumors is the situation even worse, because the 5-years survival of patients with diagnosed glioblastoma is only 2 to 3%.

#### 3.2.1. Penetration of Copolymers

**Figure 1:**
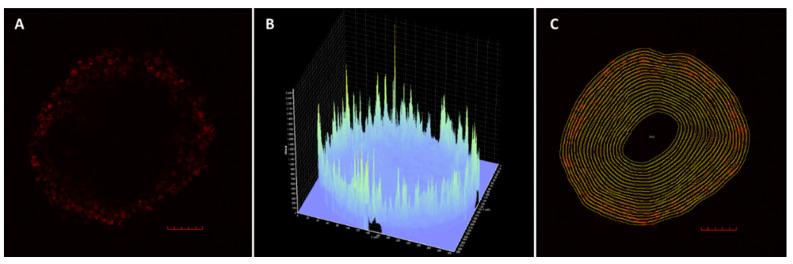
(**A**) Z stack image of a spheroid from U87-MG cells obtained by laser scanning confocal microscopy after 24 h incubation with **S1-Dy**. (**B**) The uneven 3D fluorescence intensity profile of **S1-Dy** within the U87-MG spheroid. (**C**) An illustration of the image analysis of a cross-section of the U87-MG spheroid obtained after 24 h incubation with **S1-Dy**. Z stack image through the middle of the spheroid was divided into identical layers with a width of around 9 µm. The fluorescence intensity was specified in every marked area and the maximum fluorescence intensity was determined to be 100%. This method of image analysis was used to analyze all data. The program ImageJ was used for the analysis of images. Scale bar 100 µm.

The presence of the fluorescent dye Dy-395XL covalently bound to the polymer carrier allowed the observation of the localization of copolymers within the spheroids. The fluorescently labeled polymers were incubated with spheroids and the distribution of fluorescence intensity in the center cross-section of spheroid after 24 h was analyzed from more than three images for each polymer. Due to the heterogeneous decrease in fluorescence intensity with increasing depth (Figure 1A,B), the concentric layers from surface to the center with constant width were drawn in each spheroid image (Figure 1C) and used for the analysis of fluorescence in every drawn layer to quantify the distribution of polymers within the spheroids.

**Figure 2:**
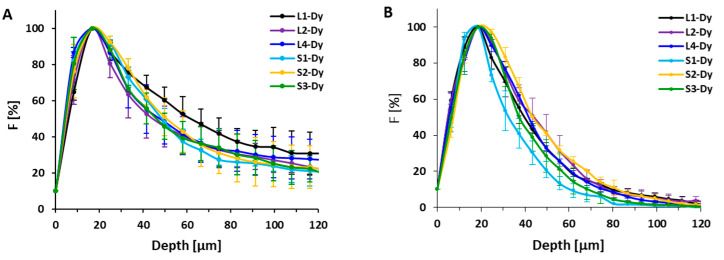
(**A**) Fluorescence profiles of fluorescently labeled HPMA-based polymer systems in C26 tumor spheroids after 24 h incubation, (**B**) fluorescence profiles of fluorescently labeled HPMA-based polymer systems in U87-MG tumor spheroids after 24 h. The fluorescence intensity dependence on the distance from the surface of the tumor spheroid was analyzed from several spheroids (>3). The percentage of fluorescence distribution is normalized to the maximum mean fluorescence intensity of the dye near the surface (100%) and outside the spheroid (0%).

The image analysis showed that all trends of the penetration of labeled copolymers without THP were similar for both spheroid types. In general, linear HPMA copolymers penetrated within both types of spheroids slightly more than HMW star-like polymers containing either biodegradable bis-MPA dendritic structure or nondegradable PAMAM dendrimer structure in the core, but the differences were not significant (Figure 2). Accumulation of all polymers peaked at 20 μm depth, then decreased up to the center of the spheroids. The depth of penetration for all polymers was more restricted in the case of U87-MG spheroids, where the fluorescence of the polymers was only detected up to 80 μm (Figure 2B), while for C26 spheroids, more profound penetration within the spheroids was observed, up to a depth of 120 μm. The difference in penetration is most probably related to the spheroid integrity since U87-MG are highly aggressive and rapidly growing cells, while the growth and proliferation of C26 cells are much slower, as evident in the rate of spheroid formation. The U87-MG cells created spheroids in three days with a diameter up to 550 µm, while the formation of spheroids from C26 cells with a diameter of around 300 µm took seven days. The differences in the proliferation rate lead to the formation of spheroids with different junctions between cells and extracellular porosity [27], resulting in different polymer penetration. For all polymers, neither the molecular weight nor dispersity caused any remarkable difference between the penetration into both types of spheroids, with partly significant differences only in the C26 spheroids in deeper penetration points. Importantly, the biodegradability of the polyester bis-MPA dendritic did not play a significant role in spheroid penetration.

**Figure 3:**
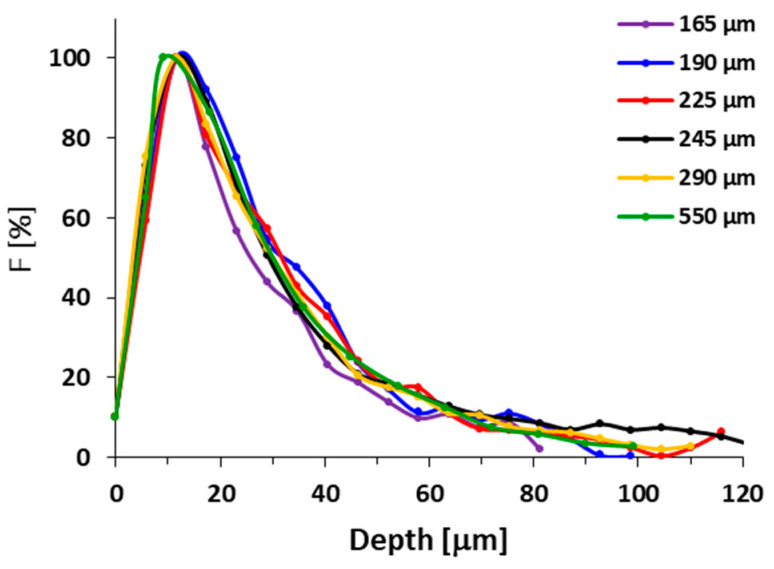
Fluorescence profiles of fluorescently labeled **L1-Dy** conjugate in U87-MG tumor spheroids of different diameters after 24 h.

Recently, the importance of the physical aspect of molecules that penetrate within spheroids was documented for PAMAM dendrimers, where larger branched structures with a higher degree of generation penetrated spheroids less than smaller dendrimers [28]. Surprisingly, we have not observed such significant differences in penetration profile between linear HPMA polymers and star-like polymers with diameters between 8 nm and 25 nm, respectively. The different penetration of non-coated PAMAM dendrimers is ascribed to their diverse surface properties of each dendrimer generation enabling strong interaction with the surrounding biological environment. However, the HPMA copolymers are known as highly biocompatible and unfolding biomaterials [29,30], therefore the structure-independent penetration into the spheroid might be the consequence of the very low interaction of copolymers with cells, which enables copolymer migration in the extracellular matrix to the deeper spheroid parts. Therefore, we can conclude that HPMA-based polymer systems enter the tumor spheroids with similar speed regardless of the hydrodynamic size in the range 8 to 25 nm. Similarly, no difference in the penetration rate of 30 nm and 50 nm poly(styrene) nanoparticles into HCT116 spheroids was observed [31]. Indeed, there was no difference in the penetration of polymer for various spheroid sizes of U87-MG spheroids (Figure 3), thus the spheroids of various sizes between 165 and 550 µm do not differ in cell density.

The benefit of macromolecules in anticancer therapy is their improved accumulation in the tumor due to the EPR effect [32], which leads to the preferable accumulation of HMW, i.e., star, polymer systems. Importantly, the penetration observations indicate that the superior anticancer effect of the star-like polymers systems is not only due to the enhanced EPR effect-based accumulation but also due to their subsequent penetration into tumor tissue remote from the blood capillaries. Although the star-like carriers are larger, they do penetrate deep in the spheroid structure comparable to the small linear counterparts. Therefore, the star-like HPMA polymer systems with a biodegradable bis-MPA core seem to be ideal candidates for following the deep preclinical investigation as they facilitate the elimination of copolymers after cargo delivery and exhibit the most convenient properties, not only in terms of tumor accumulation but also adequate penetration capability.

#### 3.2.2. Penetration of THP

**Figure 4:**
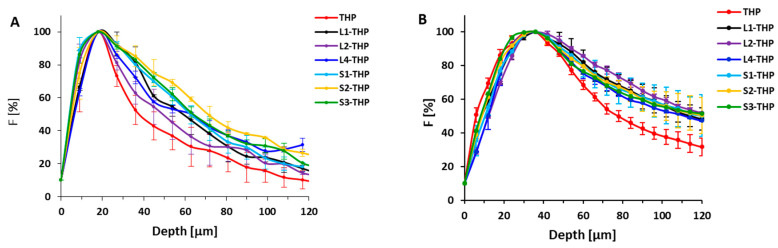
(**A**) Fluorescence profiles of THP and THP-polymer conjugates in C26 tumor spheroids after 6 h incubation and (**B**) fluorescence profiles of free THP and THP-polymer conjugates in U87-MG tumor spheroids after 6 h. The fluorescence intensity dependence on the distance from the surface of the tumor spheroid was analyzed from several spheroids (>3). The percentage of fluorescence distribution is normalized to the maximum mean fluorescence intensity of the THP near the surface (100%) and outside the spheroid (0%).

To further validate conjugate penetration data, the spheroid studies were also performed with THP-polymer conjugates in a similar setting as mentioned above. The distribution of THP within the spheroid was evaluated and the dependence of fluorescence intensity of THP fluorescence on the distance from the spheroid surface was plotted (Figure 4). This study employed HPMA copolymers with THP attached via a hydrazone bond, pH-stimuli-sensitive spacer (Table 3). The THP fluorescence was monitored by laser scanning confocal microscopy after 6 h when the cytotoxic effect of THP was less pronounced and the experiment was not influenced dramatically by the cytotoxicity of the evaluated polymer conjugates.

Importantly, for both types of spheroids, the lowest penetration was observed for the free THP due to its rapid internalization by the cells on the spheroid surface, thus preventing further drug penetration into deeper layers. Recently, a highly profound internalization of free THP in contrast to polymer-bound THP was shown [15]. The attachment of THP to copolymers resulted in more profound penetration, especially deeper penetration into the U87-MG spheroids was unambiguously confirmed. While the penetration of THP from the conjugates in C26 spheroids was comparable to the penetration of the given copolymers, the THP penetration in U87-MG spheroids was more pronounced than the penetration of the corresponding carrier, compare Figure 2 and Figure 4.

In the C26 spheroids, the hydrodynamic size, dispersity, or type of polymer carrier did not affect the penetration depth of THP originally attached to the polymer precursor. This almost identical penetration may be attributed to the penetration of the whole conjugates, which are randomly entrapped by endocytosis during penetration, and THP is then released due to the drop in pH after cellular uptake and intercalated into the cell DNA. Thus finally, both the fluorescence signal from the copolymer and THP are similarly distributed throughout the spheroid. However, the lower pH in the spheroid extracellular microenvironment [33] could cause a partial release of THP from the carrier. Hence the penetration of free THP was found to be shallower and penetration of polymer precursors and THP bound to conjugates is almost identical, we hypothesize that the release of THP in extracellular space is not present and the polymer-THP conjugate can deliver the drug to the cells in deeper layers.

By contrast, penetration of THP in the U87-MG spheroid showed different behavior, as the THP from the conjugates penetrated deeper into the spheroids compared to the corresponding copolymer. Recently, cellular transfer of polymer systems and free drugs by exosomes has been described in detail proving the cellular ability to transfer polymer cargo between cells [34]. For the pH-sensitive polymer conjugates, it was postulated that the drug is liberated upon exposure to the low pH in endosomes, then transferred as a free drug via exosomes to other cells. We hypothesize that a similar course can occur in the case of penetration of pH-sensitive THP-polymer conjugates in U87-MG spheroids, which are known to form exosomes massively [35]. The endocytosed polymer conjugate is freed of THP, then transferred via exosomes to invade the next layer of the cells in the spheroid, thereby leading to the more pronounced penetration based on the combined penetration of bound and released THP.

One of the assumptions of successful treatment is the ability of the polymer carrier to penetrate the tumor as far as possible to reach the maximum number of the tumor cells. THP bound to the polymer carrier via a hydrazone bond takes advantage not only of the EPR effect-based enhanced accumulation in the tumor tissue but also from deeper penetration into the non-vascularized tumor tissue model. Thus, the delivery of pH-sensitive bound drugs is based on complementary effects. Importantly, the increased polymer nanotherapeutics size from 8 to 25 nm, which is a prerequisite for the enhanced passive accumulation in solid tumors, did not cause any restriction in penetration, thus validating the overall applicability of HMW systems for drug delivery.

### 3.3. Cytotoxicity

Repeatedly, it was confirmed that the in vitro cytotoxicity of the free anticancer agent against cells or spheroids is higher than for its pH-sensitive conjugates with polymeric carriers [9,14,15]. This decrease of in vitro cytotoxicity is attributed to the fact that covalently bound drug in nanomedicines is in an inactive form and its cytotoxicity depends on the release rate from polymer precursor, while the free drug rapidly penetrates the cells by diffusion, acting directly on the cells. Nevertheless, the main advantage of nanomedicines relies on reduced side effects and highly improved pharmacokinetics.

Cytotoxicity on the cells in 2D and cell spheroid 3D systems in the presence of the tested THP-polymer conjugates and free THP was performed using the MTT assay and the determined IC_50_ values are summarized in Table 4. Surprisingly, the cytotoxicity of the star-like nanomedicines regardless of the inner structure was comparable to the free THP, while linear nanomedicines showed 5–6 times lower cytotoxicity, possibly because the star-like polymers carry more THP molecules than the linear polymers, thus exposing the cells to a larger cargo of drug after endocytosis. There were no significant differences in the cytotoxicity of linear nanomedicines differing in molecular weight and dispersity.

**Figure 5:**
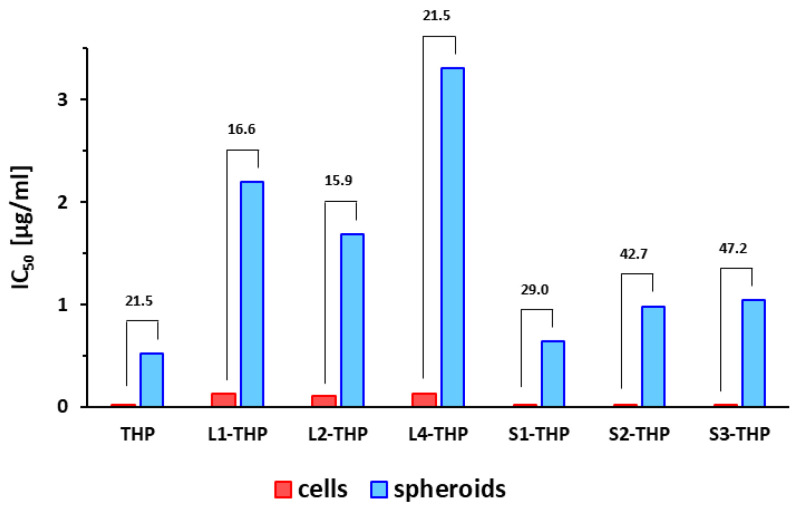
The cytotoxic effect of THP and THP-polymer conjugates on C26 monolayer cells and spheroids from the C26 cell line. The numbers above the bar graph represent the ratio of IC_50_ on spheroids and IC_50_ on 2D cells.

The IC_50_ values of nanomedicines and free THP on spheroids were around 15 to 40 times higher than in 2D tumor cell culture (Figure 5), suggesting that cells in tumor spheroids are more resistant to drugs compared to monolayer cells. The increased IC_50_ is most probably influenced by the limited accessibility of cells for used polymer nanomedicines. While the 8 nm linear nanomedicines showed a similar increase in the IC_50_ for spheroids as determined for free THP, the IC_50_ of star nanomedicines increased from cells to spheroids more significantly, almost twice compared to the linear nanomedicines and free THP. Finally, the cytotoxicity of linear and star nanomedicines is comparable on spheroids, thus, the increased polymer size did not affect the cytotoxic efficacy of HMW star nanomedicines.

These results suggest that the structure and size of nanomedicines have a minor effect on the cytotoxicity of THP-carrying nanomedicines on tumor spheroids, although differences in monolayer cell cultures were observed. Importantly, the tumor spheroid cytotoxicity of the star nanomedicines was comparable with the free drug. The superior anticancer efficacy of star nanomedicines compared to the free drug will be emphasized in vivo due to their EPR effect driven tumor accumulation and prolonged blood clearance. Thus, our results demonstrate the benefit of star nanomedicines in solid tumor therapy.

The higher IC_50_ values for C26 spheroids than for C26 cells support the hypothesis that the 2D cell models are not identical to a real tumor and may not be representative of in vivo behavior of polymer conjugates. From this point of view, the spheroid study is more reliable to estimate the in vivo activity of nanomedicines than 2D cell culture, even though the pharmacokinetics are still neglected.

## 4. Conclusions

C26 and U87-MG tumor cell spheroids were successfully employed to investigate the dependence of polymer nanomedicines penetration on the detailed structure, size, and dispersity of the systems. Importantly, there was no significant difference in penetration depth into the spheroids observed for all the studied linear and star polymers or drug THP attached to the given polymer-carriers via the pH-sensitive hydrazone bond. These nanomedicines with a size up to 25 nm can deeply penetrate spheroids, which serve as a model of non-vascularized tumor tissue surrounding the blood capillaries. Moreover, the penetration of THP applied in stimuli-sensitive nanomedicines was deeper compared to the application of free THP. Furthermore, the cytotoxicity of the studied star nanomedicines was excellent against the spheroids, almost comparable to the free THP.

Finally, it should be mentioned that the uptake into a real tumor depends on many parameters that positively or negatively influence drug transport into deeper layers of the tumor tissue as well as cells. The present study has shown that spheroids are an excellent tool for the in vitro modeling of avascular tumor tissue and a useful alternative to animal models.

## Figures and Tables

**Table 1:** Physico-chemical characteristics of *N*-(2-hydroxypropyl)methacrylamide (HPMA)-based polymer precursors.

Polymer	Type of Polymer	*M_w_* (g/mol)	*Ð*	Content of NHNH_2_ Groups ^1^ (mol%)	*D*_h_^2^ (nm)
**L1 ^3^**	linear	22,300	1.03	6.9	7.6 ± 0.29
**L2 ^3^**	linear	43,600	1.12	6.9	9.3 ± 0.60
**L3 ^3^**	linear	36,400	1.14	6.1	10.2 ± 0.9
**L4**	linear	22,200	2.09	7.0	7.8 ± 0.57
**S1**	star (bis-MPA dendron)	330,400	1.04	7.1	23.7 ± 0.56
**S2**	star (bis-MPA dendrimer)	300,000	1.05	6.8	23.0 ± 0.58
**S3**	star (PAMAM)	340,400	1.04	6.9	21.0 ± 0.17

^1^ Reactive group along the polymer chain. ^2^ Hydrodynamic diameters determined by DLS (number mean). ^3^ Functionality of main chain-end TT groups: **L2** = 0.9 and **L3** = 0.9.

**Table 2:** List of polymer-drug conjugates and their characteristics.

Conjugate	Type of Polymer Precursor	THP (wt%)	*M*_w_ of Conjugate (g/mol)	*Ð*
**L1**-THP	RAFT linear	7.08	27,600	1.22
**L2**-THP	RAFT linear	8.94	61,000	1.21
**L4**-THP	linear	9.48	26,200	1.90
**S1**-THP	star (bis-MPA dendron)	9.15	334,200	1.03
**S2**-THP	star (bis-MPA dendrimer)	10.14	306,300	1.02
**S3**-THP	star (PAMAM)	8.65	346,600	1.03

**Table 3:** List of fluorescently labeled HPMA polymer systems and their characteristics.

Conjugate	Type of Polymer Precursor	Dy-395XL (wt%)	Number of Dy/HPMA Polymer
**L1**-Dy	RAFT linear	1.6	0.6
**L2**-Dy	RAFT linear	0.7	0.5
**L4**-Dy	linear	1.3	0.3
**S1**-Dy	star (bis-MPA dendron)	1.4	1.0
**S2**-Dy	star (bis-MPA dendrimer)	1.2	1.0
**S3**-Dy	star (PAMAM)	1.6	1.0

**Table 4:** In vitro cytotoxicity of THP-polymer conjugates against monolayer colon carcinoma cells C26 and C26 spheroids detected by MTT assay.

Sample	Polymer Structure/Core	*M*_w_ (g/mol)	IC_50_ (µg/mL) ± SD	
			C26 Cells	C26 Spheroids
THP	-	-	0.024 ± 0.001	0.517 ± 0.026
**L1**-THP	linear/-	22,300	0.132 ± 0.002	2.193 ± 0.099
**L2**-THP	linear/-	43,600	0.106 ± 0.002	1.685 ± 0.108
**L4**-THP	linear/-	22,200	0.133 ± 0.004	3.303 ± 0.165
**S1**-THP	star/bis-MPA dendron	330,400	0.022 ± 0.001	0.637 ± 0.029
**S2**-THP	star/bis-MPA dendrimer	300,000	0.023 ± 0.001	0.983 ± 0.039
**S3**-THP	star/PAMAM	340,400	0.022 ± 0.001	1.038 ± 0.058

## Supplementary Materials

The following are available online at https://www.mdpi.com/1999-4923/12/12/1242/s1. Figure S1: Schematic description of the polymer carriers used in the study.

## References

[1] Minotti G., Menna P., Salvatorelli E., Cairo G., Gianni L. (2004). Anthracyclines: Molecular Advances and Pharmacologic Developments in Antitumor Activity and Cardiotoxicity. Pharmacol. Rev..

[2] Mizutani H., Hotta S., Nishimoto A., Ikemura K., Miyazawa D., Ikeda Y., Maeda T., Yoshikawa M., Hiraku Y., Kawanishi S. (2017). Pirarubicin, an Anthracycline Anticancer Agent, Induces Apoptosis Through Generation of Hydrogen Peroxide. Anticancer Res..

[3] Sugiyama T., Sadzuka Y., Nagasawa K., Ohnishi N., Yokoyama T., Sonobe T. (1999). Membrane Transport and Antitumor Activity of Pirarubicin, and Comparison with Those of Doxorubicin. Jpn. J. Cancer Res..

[4] Zhou J., Zhang X., Li M., Wu W., Sun X., Zhang L., Gong T. (2013). Novel Lipid Hybrid Albumin Nanoparticle Greatly Lowered Toxicity of Pirarubicin. Mol. Pharm..

[5] Sadatmousavi P., Chen P. (2013). Self/Co-Assembling Peptide, EAR8-II, as a Potential Carrier for a Hydrophobic Anticancer Drug Pirarubicin (THP)—Characterization and in-Vitro Delivery. Int. J. Mol. Sci..

[6] Tsukigawa K., Liao L., Nakamura H., Fang J., Greish K., Otagiri M., Maeda H. (2015). Synthesis and therapeutic effect of styrene-maleic acid copolymer-conjugated pirarubicin. Cancer Sci..

[7] Yang J., Kopeček J. (2017). The light at the end of the tunnel—Second generation HPMA conjugates for cancer treatment. Curr. Opin. Colloid Interface Sci..

[8] Islam W., Fang J., Etrych T., Chytil P., Ulbrich K., Sakoguchi A., Kusakabe K., Maeda H. (2018). HPMA copolymer conjugate with pirarubicin: In vitro and ex vivo stability and drug release study. Int. J. Pharm..

[9] Nakamura H., Etrych T., Chytil P., Ohkubo M., Fang J., Ulbrich K., Maeda H. (2014). Two step mechanisms of tumor selective delivery of *N*-(2-hydroxypropyl)methacrylamide copolymer conjugated with pirarubicin via an acid-cleavable linkage. J. Control. Release.

[10] Nakamura H., Koziolová E., Etrych T., Chytil P., Fang J., Ulbrich K., Maeda H. (2015). Comparison between linear and star-like HPMA conjugated pirarubicin (THP) in pharmacokinetics and antitumor activity in tumor bearing mice. Eur. J. Pharm. Biopharm..

[11] Chytil P., Koziolová E., Etrych T., Ulbrich K. (2018). HPMA Copolymer-Drug Conjugates with Controlled Tumor-Specific Drug Release. Macromol. Biosci..

[12] Etrych T., Strohalm J., Chytil P., Říhová B., Ulbrich K. (2011). Novel star HPMA-based polymer conjugates for passive targeting to solid tumors. J. Drug Target..

[13] Kostková H., Schindler L., Kotrchová L., Kovář M., Šírová M., Kostka L., Etrych T. (2017). Star Polymer-Drug Conjugates with pH-Controlled Drug Release and Carrier Degradation. J. Nanomater..

[14] Kostka L., Kotrchová L., Šubr V., Libánská A., Ferreira C.A., Malátová I., Lee H.J., Barnhart T.E., Engle J.W., Cai W. (2020). HPMA-based star polymer biomaterials with tuneable structure and biodegradability tailored for advanced drug delivery to solid tumours. Biomaterials.

[15] Nakamura H., Koziolová E., Chytil P., Etrych T., Haratake M., Maeda H. (2019). Superior Penetration and Cytotoxicity of HPMA Copolymer Conjugates of Pirarubicin in Tumor Cell Spheroid. Mol. Pharm..

[16] Ulbrich K., Etrych T., Chytil P., Jelínková M., Říhová B. (2004). Antibody-targeted Polymer–doxorubicin Conjugates with pH-controlled Activation. J. Drug Target..

[17] Tannenbaum J., Bennett T. (2015). Russell and Burch’s 3Rs Then and Now: The Need for Clarity in Definition and Purpose. J. Am. Assoc. Lab. Anim. Sci..

[18] Lazzari G., Couvreur P., Mura S. (2017). Multicellular tumor spheroids: A relevant 3D model for the in vitro preclinical investigation of polymer nanomedicines. Polym. Chem..

[19] Lovitt C., Shelper T., Avery V. (2014). Advanced Cell Culture Techniques for Cancer Drug Discovery. Biology.

[20] Randárová E., Nakamura H., Islam R., Studenovský M., Mamoru H., Fang J., Chytil P., Etrych T. (2020). Highly effective anti-tumor nanomedicines based on HPMA copolymer conjugates with pirarubicin prepared by controlled RAFT polymerization. Acta Biomater..

[21] Lidický O., Klener P., Machová D., Vočková P., Pokorná E., Helman K., Mavis C., Janoušková O., Etrych T. (2020). Overcoming resistance to rituximab in relapsed non-Hodgkin lymphomas by antibody-polymer drug conjugates actively targeted by anti-CD38 daratumumab. J. Control. Release.

[22] Chytil P., Etrych T., Kříž J., Šubr V., Ulbrich K. (2010). N-(2-Hydroxypropyl)methacrylamide-based polymer conjugates with pH-controlled activation of doxorubicin for cell-specific or passive tumour targeting. Synthesis by RAFT polymerisation and physicochemical characterisation. Eur. J. Pharm. Sci..

[23] Koziolová E., Kostka L., Kotrchová L., Šubr V., Konefal R., Nottelet B., Etrych T. (2018). *N*-(2-Hydroxypropyl)methacrylamide-Based Linear, Diblock, and Starlike Polymer Drug Carriers: Advanced Process for Their Simple Production. Biomacromolecules.

[24] Etrych T., Mrkvan T., Chytil P., Koňák Č., Říhová B., Ulbrich K. (2008). *N*-(2-hydroxypropyl)methacrylamide-based polymer conjugates with pH-controlled activation of doxorubicin. I. New synthesis, physicochemical characterization and preliminary biological evaluation. J. Appl. Polym. Sci..

[25] Mosmann T. (1983). Rapid colorimetric assay for cellular growth and survival: Application to proliferation and cytotoxicity assays. J. Immunol. Methods.

[26] Tomalova B., Sirova M., Rossmann P., Pola R., Strohalm J., Chytil P., Cerny V., Tomala J., Kabesova M., Říhová B. (2016). The structure-dependent toxicity, pharmacokinetics and anti-tumour activity of HPMA copolymer conjugates in the treatment of solid tumours and leukaemia. J. Control. Release.

[27] Goodman T.T., Olive P.L., Pun S.H. (2007). Increased nanoparticle penetration in collagenase-treated multicellular spheroids. Int. J. Nanomed..

[28] Bugno J., Poellmann M.J., Sokolowski K., Hsu H., Kim D.-H., Hong S. (2019). Tumor penetration of Sub-10 nm nanoparticles: Effect of dendrimer properties on their penetration in multicellular tumor spheroids. Nanomed. Nanotechnol. Biol. Med..

[29] Janisova L., Gruzinov A., Zaborova O.V., Velychkivska N., Vaněk O., Chytil P., Etrych T., Janoušková O., Zhang X., Blanchet C. (2020). Molecular Mechanisms of the Interactions of *N*-(2-Hydroxypropyl)methacrylamide Copolymers Designed for Cancer Therapy with Blood Plasma Proteins. Pharmaceutics.

[30] Klepac D., Kostková H., Petrova S., Chytil P., Etrych T., Kereïche S., Raška I., Weitz D.A., Filippov S.K. (2018). Interaction of spin-labeled HPMA-based nanoparticles with human blood plasma proteins—The introduction of protein-corona-free polymer nanomedicine. Nanoscale.

[31] Tchoryk A., Taresco V., Argent R.H., Ashford M., Gellert P.R., Stolnik S., Grabowska A., Garnett M.C. (2019). Penetration and Uptake of Nanoparticles in 3D Tumor Spheroids. Bioconjug. Chem..

[32] Kostka L., Etrych T. (2016). High-Molecular-Weight HPMA-Based Polymer Drug Carriers for Delivery to Tumor. Physiol. Res..

[33] Anderson M., Moshnikova A., Engelman D.M., Reshetnyak Y.K., Andreev O.A. (2016). Probe for the measurement of cell surface pH in vivo and ex vivo. Proc. Natl. Acad. Sci. USA.

[34] Gunár K., Kotrchová L., Filipová M., Krunclová T., Pola R., Randárová E., Etrych T., Janoušková O. (2020). The transmission and toxicity of pHPMA copolymer-bound doxorubicin containing exosomes derived from adherent two-dimensional human breast adenocarcinoma cell line and three-dimensional spheroids. Nanomed. Nanotechnol. Biol. Med..

[35] Chun S., Ahn S., Yeom C.-H., Park S. (2016). Exosome Proteome of U-87MG Glioblastoma Cells. Biology.

