# Supplementary material for: Structure-to-Efficacy Relationship of HPMA-Based Nanomedicines: The Tumor Spheroid Penetration Study
Source: Pharmaceutics. 2020 Dec 20;12(12):1242. doi: 10.3390/pharmaceutics12121242 (PMC7766879; doi:10.3390/pharmaceutics12121242)
Supplement: Supplementary file 1 [file pharmaceutics-12-01242-s001.pdf]

pharmaceutics

MDPI

Supplementary Materials: Structure-to-Efficacy Relationship of HPMA-Based Nanomedicines: The Tumor Spheroid Penetration Study

Júlia Kudláčová, Lenka Kotrchová, Libor Kostka, Eva Randárová, Marcela Filipová, Olga Janoušková, Jun Fang, Tomáš Etrych

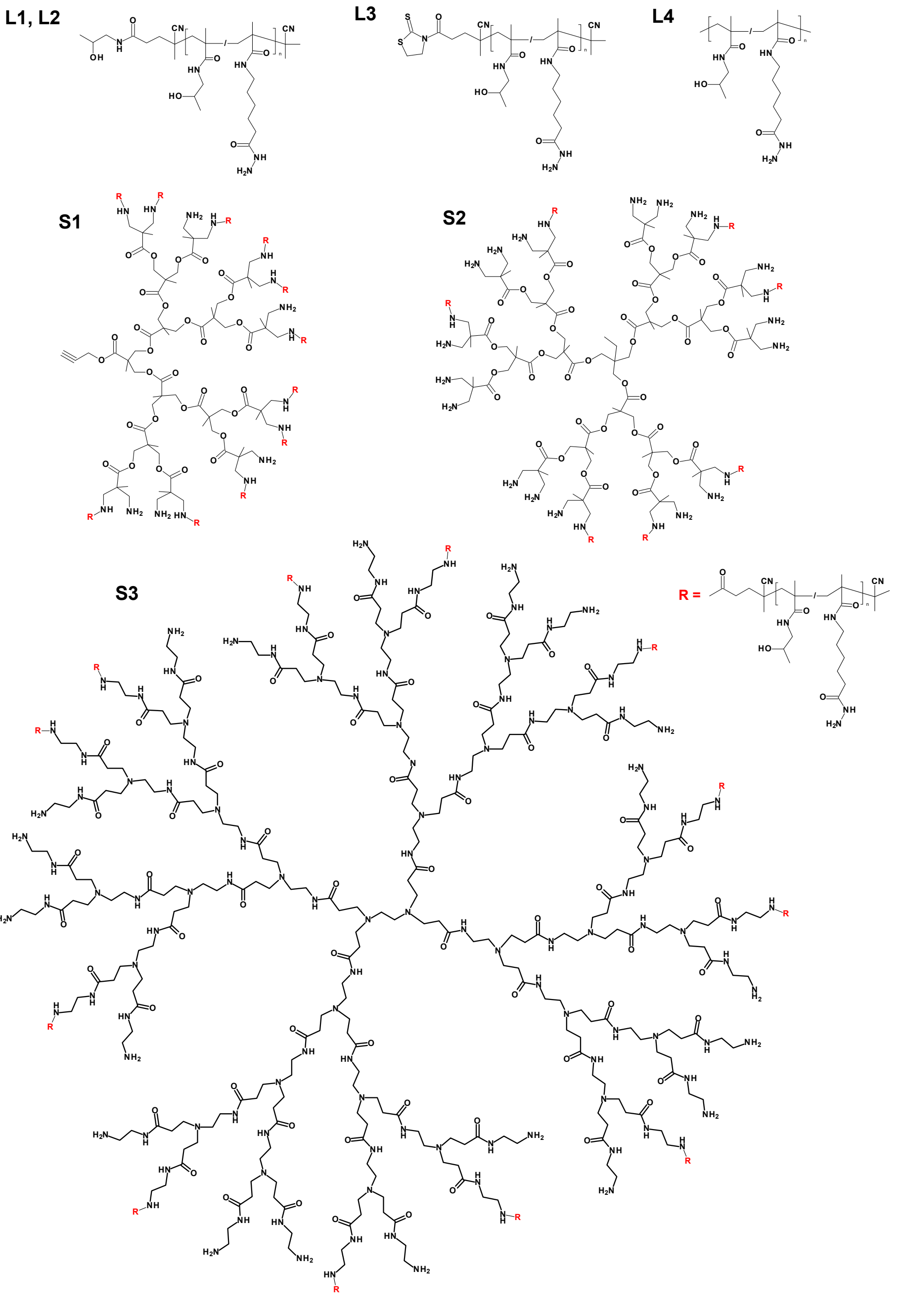

Figure S1. Schematic description of the polymer carriers used in the study.
